# Infantile Indigestion1Read at the thirty-eighth annual meeting of the Medical Association of Central New York, held at Buffalo, October 24, 1905.

**Published:** 1906-03

**Authors:** A. A. Young

**Affiliations:** Newark, N. Y.


### Infantile Indigestion.[1]

By A. A. YOUNG, M. D., Newark, N. Y.

I HAVE chosen the foregoing title for this paper because under it can be classed both conditions so frequently found,—namely, infantile diarrhea and infantile constipation. Observation has led me to believe, though widely differing in outward appearances, that these obscurable conditions are at least twins, if not even more closely related. Both spring from the same source, from the same excitants, which are usually improper food, improperly prepared, improperly given at improper hours; and to this must be added the medicaments with which the child is usually stuffed almost from its first breath.

Microorganisms may be important elements, but for their development there must be first a lowering of the vital forces, to-

1. Read at the thirty-eighth annual meeting of the Medical Association of Central New York, held at Buffalo, October 24, 1905.

gether with a suitable medium, usually imperfectly digested food within the intestinal canal, before the growth of the micro-organisms can take place. It is one of the imperative duties of the physician, beginning with the child's first outcry, to see that the helpless infant does not become the victim of all the whims and fancies of neighbors, friends, or relatives, and in country practice at least this is no easy task.

In spite of the physician's earnest protest, many times before he leaves the mother and child, most riduculous and disgusting customs are carried out. In one instance before the baby had gone through, for the first time, the process of lavage, three tea-spoonfuls of whiskey had been given it, and in less than three months the child died from malnutrition, never having been well from birth; and the family and friends are doubtless still wondering, as at the time, why the Lord took their darling! In another case, not mine, the child had just been clothed, a marked stir among the women was observed and the question was asked, "where is the father?" the doctor asks, "what do you want of the father? The child and mother are all right?" "Doctor," came the reply, "don't you know if we do not find the father and get some of his urine for the baby to drink it may be a fool?" I could multiply similar illustrations but time will not permit. Similar proceedings some of you may have encountered, and may, too, have learned the seriousness of objecting to womens whims.

Doubtless all are familiar with the "home remedies," anise seed tea, catnip tea, saffron tea, soothing syrup, cordials, and the like, with which the child must be doped from the very first day of its existence, on account of its inherited imperfections due to the sins of Adam, though these imperfections are not apparent to the physician. To prevent this lay prescribing is the physician's first serious obstacle; for to oppose a mother, or her feminine advisors, is to invite and receive most severe criticism if not subsequent dismissal. We must admit that such interference with the child's normal digestive apparatus is too often allowed while it should have been unqualifiedly condemmed. After the first care of the baby, attention should be given to the mother's milk, the incomparable baby food. Does it contain the proper ingredients, and are they in the proper proportions, and does it contain any abnormal material? To this question the degree of the child's nourishment and growth is quite a sure index.

Milk, so far as a dietetic is concerned, is only a mixture and therefore requires not the services of an expert chemist to determine approximately the amount of each ingredient entering into it. If there be a deficiency in one or more of the normal ingredients, supply it, if possible, through the mother's food; if there be an overplus, reduce it by similar measures. The quantity and character of human milk must be influenced by surrounding conditions the same as in the lower order of animals, as by fright, or even the presence of strangers during the milking period. Alimentation, too, so far as the cow is concerned, plays no unimportant part; the clover, the pumpkin, the cabbage, the refuse from farms as well as the silo impart to the milk products a characteristic odor and a characteristic taste which are indicative of an altered condition of the milk.

For purposes of illustration, I will cite a case that came under my observation. The patient, a baby, aged three weeks, contracted diarrhea; it was bottle fed from birth with milk supplied from the cow owned by the family. For the diarrhea, the ordinary remedies were prescribed but no satisfactory results followed. Inquiry was then made as to the milk supply and it appeared that the cow was a Jersey; that it had good pasturage, and also had a liberal supply of "gluten food" in addition thereto. It was evident, then to me, that the diarrhea was dependent upon the changed relation of the component parts of the milk given, making it, for the child, indigestible but of itself not unwholesome or impure; no degree of Pasteurisation could have improved this milk. It was then ordered that the gluten food be discontinued, with all other refuse matter save good pasturage, good water, and an ample supply of salt. Immediately following this change the child began to improve and made a rapid recovery and practically without medical aid.

Another predisposing cause of infantile indigestion is overfeeding, both in frequency and amount, whether such food comes from the mother's breasts, or from the bottle. I believe the infants are few and far between who can consume from four to six ounces of milk for each meal, every two hours, without contracting some intestinal affection; this amount of milk for the child, means to the adult, nearly two quarts for each meal. To force such an amount into an adults stomach would make him think, in harmony with his mother-in-law, that he has worms, like interested friends so often see in babies and who are then so kind as to advise the physician what is the matter, and the medicine to be used. There is no organ of the body but that requires nearly as much rest as labor to maintain its healthful state. The healthful state of each organ requires its rest to be commensurate with the labor performed.

It is generally conceded that to complete stomach digestion, even of milk, requires nearly two hours, and if nourishment be given thus frequently there is no time for rest, no time for the glandular structures to produce and excrete more of the complex digestive fluid; then organic action ceases from exhaustion. Time for digestion, and rest after digestion, are two important factors in alimentation. Robbed of time and rest, abnormal conditions will surely follow. Let me here add that the occular abnormal condition, diarrhea, is not a disease of itself, but it is nature's method and effort to expell decaying or germ infected material from the alimentary canal; check our theoretical germ-development by elimination of abnormal food products and the disease is at an end. Constipation, diarrhea's mate, requires a similar treatment,—the expulsion of retained germ-infected feces which produce a paralysing influence upon the mucosa and muscular structures of the intestinal tract.

It is to be inferred, that the non-infected feces, undergoing the process of normal digestion are healthful products and as such are in a certain degree germicides. Nature needs no extra aid in separating the nutriment material from the refuse material and in eliminating the latter from the body when this condition exists. In all cases of infantile indigestion, whether it appears as diarrhea, constipation, or obstipation, the first duty of the physician is to clear the alimentary canal of all germ-infected material; the agent or agents to be employed must depend upon the individual judgment of the prescriber. Shall he depend upon digestants, upon astringents, upon antiferments, upon sedatives, upon evacuants by mouth or rectum, or any other suggested method? All methods may be good but some may be wrong when applied to an individual case. To force food into the child's stomach, under the plea of nourishment, while endeavoring to clear the intestinal tract, already overburdened from work, is to me the height of the ridiculous, for no other service can be rendered by such proceedings than the adding of fuel to the embers passed by in endeavoring to quench the active flame ahead. Withdraw all food, if you can, for a reasonable season, and let the "rest cure" to do its legitimate work.

Allow me here to mention an illustrative case. A baby, a little more than a year old, had a severe but typical attack of infantile diarrhea; the case was seen early and those medicinal agents best suited for the child were prescribed. In connection with medicaments the withholding of all foods was ordered,—unless water be a food. The child grew gradually worse and it was inferred that the orders of the physician were not fully carried out. This surmise was corroborated by a neighbor who informed me that the child was being fed at unreasonable hours and with unreasonable things. An accusation from this neighbor to the family brought forth an emphatic denial with the remark, "We are doing just as the doctor said." While this conversation was going on, the child vomited the remains of an entire molasses cooky given only a short time before. Upon my arrival the next day the mother met me at the door and remarked, "the baby is most gone and I thought I would not disturb it any more but let it die in peace." The condition in which the baby was found is better imagined than described. Suffice to say the use of soap and water was ordered for immediate use accompanied by the remark, "if this baby goes to Heaven it will go a clean baby." I had previously learned of the cooky episode, so orders were left as to dietetics, in no uncertain language. The child then began to improve and was soon restored to health with but little medicament.

I am satisfied that much mischief has been done, many little mounds added to "God's Acre" by following out that once popular slogan, "Nourishment," "Nourishment," which only meant more work for an already overworked digestive apparatus, more culture material for increased microorganic growths, more local and systemic infection for the patient. It is evident that there must be a depraved or altered condition of the human organism prior to the deposition and growth of specific microorganisms, or at least a cessation of functional activity of one or more organs of the body.

Accepting this theory, then, the first indication for treatment is prophylaxis,—to begin prescribing, not medicinal agents, but advice prior even to the birth of the child. The prospective parents should be informed as to child dietetics, so that beginning with its arrival it may be cared for in a reasonable way as nature intended. Why should the new born child with its first outcry be called ill and be doped with anise seed tea, saffron tea, whiskey, nannyberry tea, or human urine? The outcry at birth, and following is, as is well known, a normal physiological process and its evident intent is to stimulate physical processes or actions necessary to begin and sustain infantile life. Why should the child be called hungry, and forced feeding be enjoined, because intuitively the child's thumb finds its way to its mouth? This is also a physiological or normal action, the evident purpose of which is to stimulate some glands to activity, the substances from which are essential in the alimentary canal to prepare it for digestive activity. Let the child's keeper, for it only knows at birth how to cry and suck, be thoroughly enjoined from giving anything save that suggested by nature, nature's food milk and water.

I personally can see no reason why the infant should become more susceptible to intestinal diseases than the adult, if common sense on the part of parents and friends be exercised. Why, I ask, should the child's body in which there is but little waste, need food oftener than an adult who leads a strenuous life? Strenuosity and frequent eating, we all know, do not well agree and if injurious to the adult, how much more injurious to the infant, in whom there is but little waste and such waste consists largely in the evaporation or elimination of water, which substance can readily be replaced without taxing the digestive organs directly or indirectly. Prophylaxis is our most difficult problem in infantile indigestion, for it deals with something that does not exist, but it is none the less important for all that. Prophylaxis can only be administered through the infant's sponsor, a personage too often irresponsible, egotistical, and irrepressible. On account of these characteristics, the physician's instructions are often entirely ignored till intestinal manifestations become serious; then the doctor is quickly urged to do something to save the child, when perhaps it is already unsavable. It is especially true in infantile life that an ounce of prevention is worth pounds of cure and that prevention is the common sense care of the baby, a restorative and preventive human beings should have at hand.

A word in reference to medical treatment. It seems to be an acknowledged fact that infantile indigestion is the result of improper alimentation. Granting this, a differential as to excitants can readily be made and a suitable treatment instituted. In most of the cases which have come under my observation, undigested milk casein appeared to be the excitant; its prevention, and when present its elimination, is desirable. The use of opium, or any of its derivatives, I most unqualifiedly condemn; while they relieve pain, giving temporary comfort, they leave within the intestinal canal the same irritants which often act with renewed violence after the effects of the opium has passed. Antiseptics may be beneficial, in a way, but I surmise that their continued use will do harm by decreasing the activity of the digestive ferments with which they may come in contact, and aggravate the condition they are intended to restore.

The administration of cathartics, or laxatives, may be good but if followed by the taking of food while the intestinal tract is yet irritable, old conditions are likely to be restored and with renewed activity; after catharsis give the digestive organs a commensurate rest. In a case of infantile indigestion the first object sought is the elimination of all material within the rectum and colon which is usually readily accomplished by the use of an enema, to which creolin or other antiseptic has been added. The lower bowel once cleaned allows the retained matter from above to pass downward and take the place of the expelled contents which may in turn be expelled also. The next procedure is an attempt to convert the solids, of the intestinal canal into the semisolids, thus preventing impaction and favoring their elimination. For this purpose nothing has served me better than purified manna, and since milk is so frequently deficient in lactose, this too is added.

For convenience in administration tablets are made for me containing about 5 grains each of manna and lactose. If an antacid be desired, to the above named ingredients can be added milk of magnesia or lime water advantageously. Last, but not least, as a remedial agent, comes pure water, water in abundance, internal and external; it is the child's natural food; it is the child's natural medicament. In a word, the object desired in a case of infantile indigestion is the elimination of all irritants,— imperfectly digested food is one,—and absolute rest of the digestive organs following such elimination.

22 East Miller Street.

